# Supplementary material for: HIVprotI: an integrated web based platform for prediction and design of HIV proteins inhibitors
Source: J Cheminform. 2018 Mar 9;10:12. doi: 10.1186/s13321-018-0266-y (PMC5845081; doi:10.1186/s13321-018-0266-y)
Supplement: Supplementary file 2 — Additional file 2. Source code of HIVProtI web server. [file 13321_2018_266_MOESM2_ESM.zip › HIVprotI_Source-code/hivprotI/weka-3-6-6/doc/weka/classifiers/xml/XMLClassifier.html]

XMLClassifier


---


|  |  |  |
| --- | --- | --- |
| **Overview** \| **Package** \| **Class** \| **Tree** \| **Deprecated** \| **Index** \| **Help** \| **Weka's home** | |  |
| PREV CLASS   NEXT CLASS | **FRAMES**    **NO FRAMES**     **All Classes** |  |
| SUMMARY: NESTED \| FIELD \| CONSTR \| METHOD | DETAIL: FIELD \| CONSTR \| METHOD |  |


---


## weka.classifiers.xml Class XMLClassifier

```
java.lang.Object
  weka.core.xml.XMLSerialization
      weka.core.xml.XMLBasicSerialization
          weka.classifiers.xml.XMLClassifier
```

**All Implemented Interfaces:**: RevisionHandler

---

``` public class XMLClassifier extends XMLBasicSerialization ```

This class serializes and deserializes a Classifier instance to and
fro XML.  

**Version:**
:   $Revision: 1.6 $

**Author:**
:   FracPete (fracpete at waikato dot ac dot nz)

---

**Field Summary**

**Fields inherited from class weka.core.xml.XMLBasicSerialization**

`VAL_CELLS, VAL_KEY, VAL_MAPPING, VAL_VALUE`

**Fields inherited from class weka.core.xml.XMLSerialization**

`ATT_ARRAY, ATT_ARRAY_DEFAULT, ATT_CLASS, ATT_NAME, ATT_NULL, ATT_NULL_DEFAULT, ATT_PRIMITIVE, ATT_PRIMITIVE_DEFAULT, ATT_VERSION, DOCTYPE, ROOT_NODE, TAG_OBJECT, VAL_NO, VAL_ROOT, VAL_YES`


**Constructor Summary**

`XMLClassifier()`
  
          initializes the serialization


| **Method Summary** | |
| --- | --- |
| `void` | `clear()`             generates internally a new XML document and clears also the IgnoreList and the mappings for the Read/Write-Methods |
| `java.lang.String` | `getRevision()`             Returns the revision string. |

**Methods inherited from class weka.core.xml.XMLBasicSerialization**

`readCollection, readCostMatrixOld, readDefaultListModel, readMap, readMatrix, readMatrixOld, writeCollection, writeCostMatrixOld, writeDefaultListModel, writeMap, writeMatrix, writeMatrixOld`

**Methods inherited from class weka.core.xml.XMLSerialization**

`fromXML, getVersion, main, read, read, read, read, readBooleanFromXML, readByteFromXML, readCharFromXML, readDoubleFromXML, readFloatFromXML, readFromXML, readFromXML, readIntFromXML, readLongFromXML, readShortFromXML, toXML, write, write, write, write, writeToXML`

**Methods inherited from class java.lang.Object**

`equals, getClass, hashCode, notify, notifyAll, toString, wait, wait, wait`

**Constructor Detail**

### XMLClassifier

```
public XMLClassifier()
              throws java.lang.Exception
```

:   initializes the serialization

    **Throws:**: `java.lang.Exception` - if initialization fails


**Method Detail**

### clear

```
public void clear()
           throws java.lang.Exception
```

:   generates internally a new XML document and clears also the IgnoreList and
    the mappings for the Read/Write-Methods

    :   **Overrides:**: `clear` in class `XMLBasicSerialization`
    :   **Throws:**: `java.lang.Exception` - if initializing fails

---


### getRevision

```
public java.lang.String getRevision()
```

:   Returns the revision string.

    :   **Specified by:**: `getRevision` in interface `RevisionHandler` **Overrides:**: `getRevision` in class `XMLBasicSerialization`
    :   **Returns:**: the revision


---


|  |  |  |
| --- | --- | --- |
| **Overview** \| **Package** \| **Class** \| **Tree** \| **Deprecated** \| **Index** \| **Help** \| **Weka's home** | |  |
| PREV CLASS   NEXT CLASS | **FRAMES**    **NO FRAMES**     **All Classes** |  |
| SUMMARY: NESTED \| FIELD \| CONSTR \| METHOD | DETAIL: FIELD \| CONSTR \| METHOD |  |


---
